# Micro SleepNet: efficient deep learning model for mobile terminal real-time sleep staging

**DOI:** 10.3389/fnins.2023.1218072

**Published:** 2023-07-28

**Authors:** Guisong Liu, Guoliang Wei, Shuqing Sun, Dandan Mao, Jiansong Zhang, Dechun Zhao, Xuelong Tian, Xing Wang, Nanxi Chen

**Affiliations:** ^1^Department of Biomedical Engineering, Bioengineering College, Chongqing University, Chongqing, China; ^2^Department of Sleep and Psychology, Institute of Surgery Research, Daping Hospital, Third Military Medical University (Army Medical University), Chongqing, China; ^3^School of Medicine, Huaqiao University, Quanzhou, Fujian, China; ^4^College of Bioinformatics, Chongqing University of Posts and Telecommunications, Chongqing, China

**Keywords:** sleep staging, real-time efficiency, lightweight design, deep learning, model deployment

## Abstract

The real-time sleep staging algorithm that can perform inference on mobile devices without burden is a prerequisite for closed-loop sleep modulation. However, current deep learning sleep staging models have poor real-time efficiency and redundant parameters. We propose a lightweight and high-performance sleep staging model named Micro SleepNet, which takes a 30-s electroencephalography (EEG) epoch as input, without relying on contextual signals. The model features a one-dimensional group convolution with a kernel size of 1 × 3 and an Efficient Channel and Spatial Attention (ECSA) module for feature extraction and adaptive recalibration. Moreover, the model efficiently performs feature fusion using dilated convolution module and replaces the conventional fully connected layer with Global Average Pooling (GAP). These design choices significantly reduce the total number of model parameters to 48,226, with only approximately 48.95 Million Floating-point Operations per Second (MFLOPs) computation. The proposed model is conducted subject-independent cross-validation on three publicly available datasets, achieving an overall accuracy of up to 83.3%, and the Cohen Kappa is 0.77. Additionally, we introduce Class Activation Mapping (CAM) to visualize the model’s attention to EEG waveforms, which demonstrate the model’s ability to accurately capture feature waveforms of EEG at different sleep stages. This provides a strong interpretability foundation for practical applications. Furthermore, the Micro SleepNet model occupies approximately 100 KB of memory on the Android smartphone and takes only 2.8 ms to infer one EEG epoch, meeting the real-time requirements of sleep staging tasks on mobile devices. Consequently, our proposed model has the potential to serve as a foundation for accurate closed-loop sleep modulation.

## 1. Introduction

Sleep is a crucial aspect of an adult’s life, occupying approximately one-third of their day, and playing a vital role in promoting bodily repair and energy restoration (Luyster et al., 2012). According to the survey, since the COVID-19 pandemic, many individuals experience varying degrees of sleep problems (Jahrami et al., 2021). Chronic sleep deprivation and sleep disorders have been linked to an elevated risk of multiple diseases (Medicine, 2006). Polysomnography (PSG), a multi-channel sleep monitoring device, is the gold standard for accurately diagnosing and analyzing different sleep disorders in the clinic. The American Academy of Sleep Medicine (AASM) has classified each 30-s sleep record into five stages, including W, N1, N2, N3, and REM (Chriskos et al., 2021). Professional physicians manually stage the whole-night EEG, EOG, EMG, and other electrophysiological signals recorded by PSG in accordance with AASM standards. However, manual sleep staging requires specialized knowledge and is a time-intensive process. Additionally, personal scorers may have individual scoring preferences, leading to inter-scorer variability (Rosenberg and Van Hout, 2013). These unfavorable factors make manual sleep staging inefficient and expensive.

In recent years, the application of artificial intelligence (AI) technology to improve the efficiency of manual sleep staging has become a popular research topic. Previous research has focused on the development of automatic sleep staging algorithms based on machine learning and deep learning techniques (Sarkar et al., 2022). Traditional machine learning sleep staging methods typically involve two steps: manual feature extraction and classification using a machine learning classifier. However, manual feature extraction heavily relies on specialized knowledge, which can lead to missing critical features and limit the classification performance of the classifier, resulting in low staging accuracy. Additionally, the manual feature extraction method limits the model to specific data distributions, resulting in poor transferability of the model. Recently, deep learning techniques, which emerged in the field of computer vision, have been widely used in various fields due to their powerful automatic feature extraction and nonlinear fitting capabilities. Some researchers have built deep learning models for sleep staging based on convolutional neural networks (CNN), long short-term memory (LSTM), Transformer, and others, achieving significantly better staging results than traditional machine learning (Supratak et al., 2017; Phan et al., 2019a, 2022b).

However, current deep learning sleep staging models also have some limitations. Since there is temporal information in the transitions between different sleep stages, most traditional sleep staging models use RNN-like structures to predict the staging category for a period of time based on the input signal sequence, resulting in reduced real-time efficiency (Phan et al., 2019b, 2022a; Liu et al., 2023). Therefore, these models are not suitable for high real-time sleep staging scenarios, such as real-time sleep modulation in home settings. Furthermore, RNN-like structures introduce a significant number of parameters into the model, for instance, the number of trainable parameters of DeepSleepNet is approximately 21 M (Supratak et al., 2017). It is totally unsuitable for deployment on mobile devices for wearable sleep monitoring due to limited memory and processing power. We attribute the above limitations to the fact that previous studies have not fully considered the practical application scenarios of when conducting model design, especially the practical demand for automated sleep staging based on a wide range of mobile devices, leading to the general unsuitability of the current models for mobile deployment. In addition, due to the inherent black-box characteristic of deep learning, the interpretability of the model is still an unsolved problem, which affects the trust of physicians and users in the models and severely restricts the practical clinical application.

In an attempt to improve the sleep quality of sleep-disordered individuals, real-time sleep-assist systems based on wearable devices rely on acoustic stimulation, electrical stimulation to dynamically modulate subjects during sleep in real time. Nguyen et al. (2022) designed a wearable closed-loop auditory stimulation sleep-assist system based on multichannel EEG and reduced the sleep onset latency time by more than 24 min; Lu (2020) designed a low-power Soc for stage-specific optical stimulation based on bone-conduction acoustic stimulation to assist sleep and applied optical stimulation to achieve wakefulness; Liu and Richardson (2021) designed a low-power Soc for stage-specific optical stimulation. All the above systems require real-time inference of the current sleep staging. Therefore, there is an urgent need for a lightweight, real-time, and accurate sleep staging algorithm that can be deployed on various computing power-constrained mobile hardware terminals. Obviously, most of the current deep learning sleep staging models do not fulfill the above conditions.

Based on the above limitations of previous studies, after we revisit the task requirements for wearable real-time sleep monitoring and modulation scenarios, we propose Micro SleepNet, a deep learning sleep staging model for real-time inference on mobile. Our contributions can be summarized as follows:

1. We propose a lightweight sleep staging model that utilizes one-dimensional group convolution, as well as extremely lightweight ECSA module for feature extraction and adaptive feature recalibration, and efficient feature fusion using dilated convolution. Finally, we replace the fully connected layer with GAP. The above design greatly reduces the number of parameters and the computational effort of the model.
2. We introduce CAM to visualize the model’s level of attention to EEG waveforms and provide the predicted posterior probabilities. The results show that the model accurately locates the feature waves of different EEG periods, revealing the interpretability of the model and validating the rationality of the model design. To the best of our knowledge, this is the first time that CAM has been applied to the EEG sleep staging field.
3. The proposed model is evaluated on three publicly available healthy human datasets, and the results show that, with significantly lower parameters and computational complexity than traditional deep learning models, it still achieves competitive performance. In addition, we deploy the model on an Android smartphone, with a memory footprint of about 100 KB and an inference time of about 2.8 ms per data, meeting the requirements of mobile device tasks.

The rest of this paper is organized as follows: Section 2 provides a detailed description of the design of the proposed lightweight model. The experiment design is described in the Section 3. Section 4 presents the experimental results, and Section 5 provides discussion.

## 2. Materials and methods

**Figure 1:**
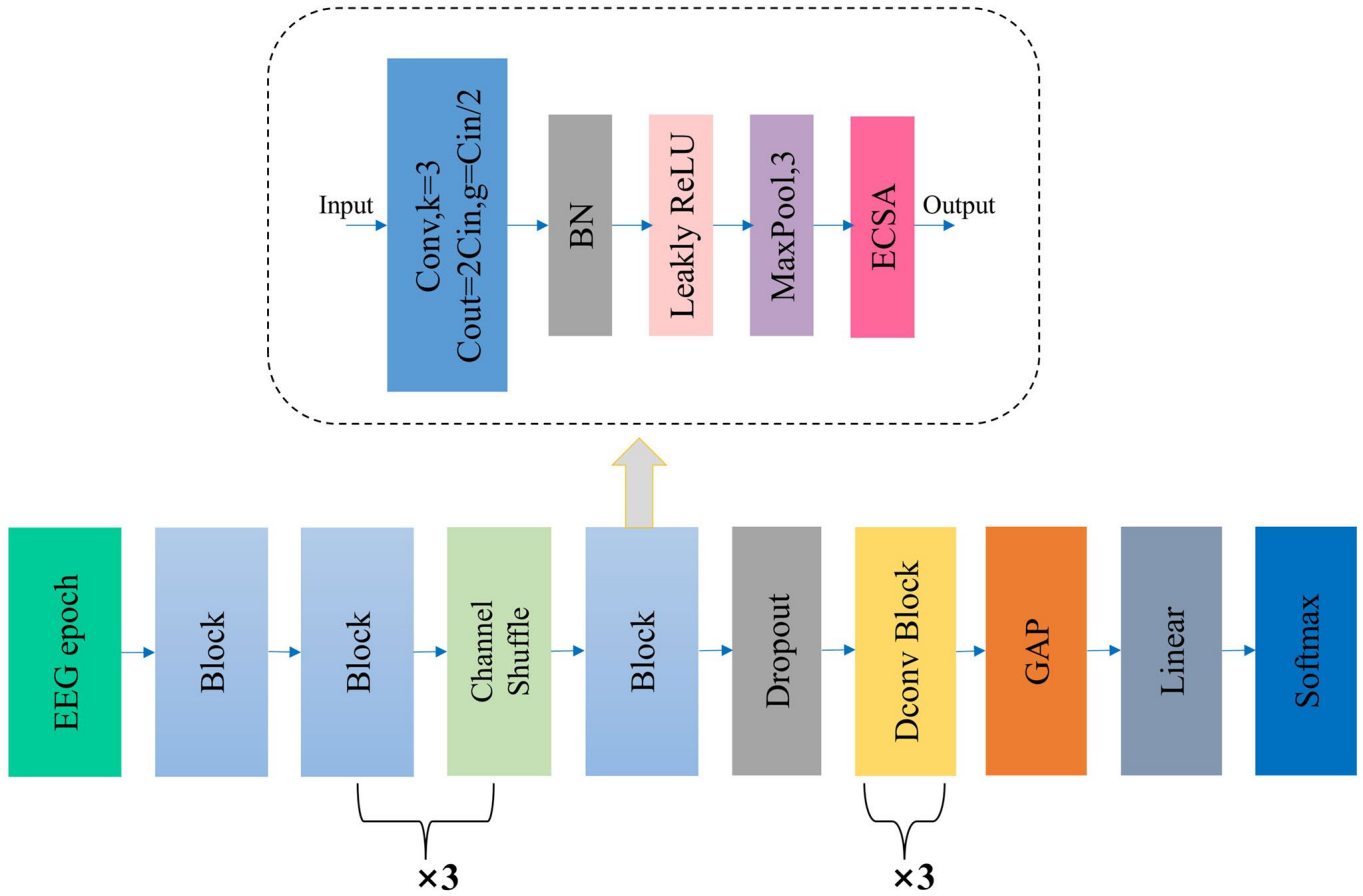
The overall structure of our proposed Micro SleepNet. Block denotes our proposed feature extraction and adaptive recalibration module, which is composed of group convolution and ECSA module, etc. The detailed structure is shown in the dashed box indicated by the arrow; Dconv Block denotes the dilated convolution module; GAP represents global average pooling. ECSA and the detailed structure of Dconv Block are shown below.

Figure 1 illustrates the overall workflow of Micro SleepNet model. The input to the model is a 30-s EEG epoch containing 3000 sampling points, corresponding to an EEG sampling rate of the common 100 Hz. The EEG epoch undergoes 5 layers of feature extraction and adaptive recalibration module with dropout. The features are then efficiently fused using 3 layers of dilated convolution modules, followed by classification using a Linear layer via GAP. The input and output dimensions of each layer of the model are shown in Table 1. The Block module consists of a group convolution and ECSA module. Each of these components is described in detail below.

**Table 1:** Details of the input and output dimensions, convolution kernel, stride and padding of each layer of the model.

Layer name	Input dim	Output dim	Kernel	Stride	Padding
Block	(B, 1, 3000)	(B, 64, 1000)	3	1	1
Block	(B, 64, 1000)	(B, 128, 333)	3	1	1
Block	(B, 128, 333)	(B, 128, 111)	3	1	1
Block	(B, 128, 111)	(B, 128, 37)	3	1	1
Block	(B, 128, 37)	(B, 128, 37)	3	1	1
Dropout	(B, 128, 37)	(B, 128, 37)	–	–	–
Dconv	(B 128, 37)	(B, 32, 37)	3	1	1
Dconv	(B, 32, 37)	(B, 64, 37)	3	2	2
Dconv	(B, 64, 37)	(B, 128, 37)	3	4	4
GAP	(B, 128, 37)	(B, 128, 1)	–	–	–
Linear	(B, 128*1)	(B, 5)	–	–	–

B is the Batch size.

### 2.1. Feature extraction and adaptive recalibration

The drawback of traditional convolutional operation is the redundancy in both parameters and computational requirements. The formulas for calculating the parameters and computational requirements, ignoring bias, are as follows:

Traditional convolutional operation parameters:

(1) $$\left(k*C_{in}*C_{out}\right)$$

Computational requirements:

(2) $$\begin{array}{c} \left(k*f_{in}*C_{in}*C_{out}\right) \end{array}$$

Where k represents the kernel size, $C_{in}$ represents the number of input feature map channels, $C_{out}$ represents the number of output feature map channels, and $f_{in}$ represents the length of the input feature map.

Inspired by ShuffleNet, we adapt one-dimensional group convolution for feature extraction (Zhang et al., 2018). Group convolution reduces the number of channels in each convolution feature map by grouping the input feature map along the channel direction, thereby reducing the parameters and computational requirements of the convolution operation. The formulas for calculating the parameters and computational requirements, ignoring bias, are as follows:

Group convolutional operation parameters:

(3) $$\begin{array}{c} \left(k*\frac{C_{in}}{g}*C_{out}\right) \end{array}$$

Computational requirements:

(4) $$\begin{array}{c} \left(k*f_{in}*C_{in}*C_{out}/g\right) \end{array}$$

Where g represents the number of groups in group convolution. It can be observed that group convolution reduces both the parameters and computational requirements of traditional convolution by a factor of g. However, this approach leads to a problem known as “neighboring multiplication” in which the convolution kernel can only merge fixed channel features in each group convolution. As a result, this weakens the exchange of information between different channel features, limiting the effectiveness of the feature extraction process. To address this issue, ShuffleNet proposed the use of channel shuffle between every two group convolution operations. In our study, we insert three channel shuffle modules within five Blocks to improve the flow of features between different layers of group convolution.

Inspired by the VGG model design philosophy, all group convolutions in our study utilize a 1 × 3 kernel size with a stride and padding of 1. Except for the first group convolution with 64 channels and *g* = 1, all other group convolutions have 128 channels and *g* = 64. The feature map size remains the same before and after the group convolution operation. Despite the increase model depth, a small kernel size of 1 × 3 can still offer a large receptive field (Simonyan and Zisserman, 2014). Additionally, small kernels further reduce the parameters and computational requirements of the model.

After each group convolution operation, we perform a batch normalization (BN) operation, which can accelerate model convergence and improve generalization (Ioffe and Szegedy, 2015). Subsequently, we apply the leaky rectified linear unit (Leaky ReLU) activation function.

(5) $$\begin{array}{c} LeakyReLU=\{\begin{array}{c} x,x\geq 0\\ \alpha x,x<0 \end{array} \end{array}$$

The Leaky ReLU function introduces a leaky value in the negative half interval, which can solve the zero gradient vanishing problems for negative values (Xu et al., 2015). Subsequently, a one-dimensional max-pooling downsampling is performed with a stride of 3, which reduces the feature map size and expands the receptive field of the convolution operation. It is important to note that downsampling is only performed in the first four blocks to provide sufficient resolution for subsequent feature fusion. This downsampling reduces the original 3000 sampling points to 37 feature points, which is equivalent to a downsampling of approximately 100 times for the original EEG data.

The effectiveness of feature extraction using one-dimensional convolution alone is limited, as the model does not prioritize different features despite extracting a large number of them from the raw EEG signal. This contradicts the doctor’s approach of manually staging sleep stages based on the waveform features of EEG signals at different times.

**Figure 2:**
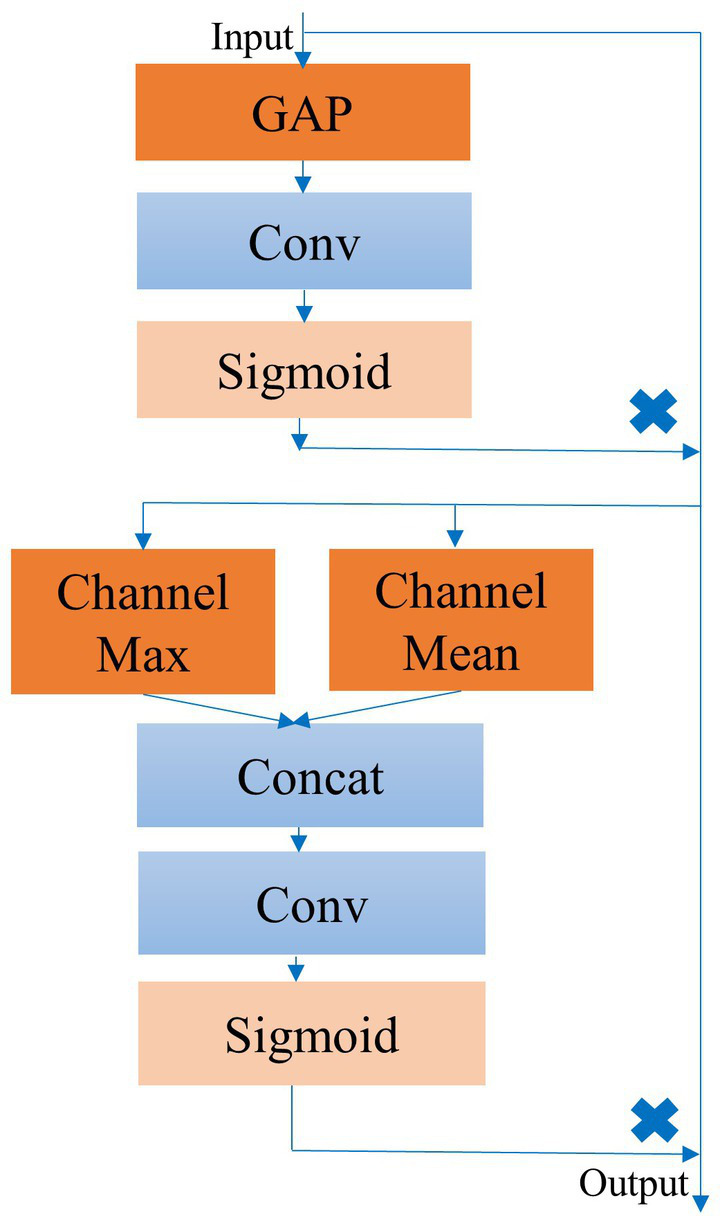
Detailed structure of the ECSA module.

To further enhance model performance, we propose the ECSA module, which we insert at the end of each Block in the feature extraction and adaptive recalibration module. Figure 2 provides a detailed schematic of this module, which consists of the Efficient Channel Attention (ECA) module and the spatial attention sub-module of the Convolutional Block Attention Module (CBAM) in series. This module has few parameters and does not change the size or number of channels of the input feature map, allowing it to be inserted into any position of the model without burden and achieve performance gains.

The ECA module is a lightweight channel attention module that builds upon the Squeeze and Excitation (SE) module (Hu et al., 2018; Wang et al., 2020). It can perform local inter-channel interaction and generate attention coefficients for the feature map channels adaptively after activation.

Specifically, the interaction range of the one-dimensional convolution is proportional to the convolution kernel k and the channel dimension C. The adaptive convolution kernel k is determined as follows:

(6) $$\begin{array}{c} k=\varphi \left(C\right)={\left|\frac{\log_{2}\left(C\right)}{\gamma }+\frac{b}{\gamma }\right|}_{odd} \end{array}$$

where the parameters γ and b are set to 2 and 1, respectively. The ECA module as a whole can be expressed as follows:

(7) $$\begin{array}{c} channelattention=Sigmoid\left(conv_{1d}\left(AvgPool\left(F_{c}\right)\right)\right) \end{array}$$

(8) $$\begin{array}{c} F_{c}=F_{c}*channelattention \end{array}$$

where $F_{c}$ is the feature map after max-pooling.

To aggregate the downsampled feature maps, we employ GAP and replace the two fully connected layers in the SE module using a one-dimensional convolution with adaptive kernels. The sigmoid activation function yields attention coefficients for each channel in the feature map, which are used to recalibrate the original feature map.

Channel attention is concerned with the contribution of different channels in the feature map to the model classification performance. In our task, this represents the contribution of different frequency components of EEG features extracted by various convolutional kernels. However, channel attention has limitations since EEG feature waves may appear at any position in the feature map, causing the contribution of spatial information within the same channel to the model classification performance to vary. Therefore, we introduce a spatial attention module by incorporating the spatial attention sub-module of the CBAM (Woo et al., 2018) after the ECA module. This further improves the ability of model to capture and focus on significant EEG waveform features in the feature maps.

Initially, the spatial attention module computes the maximum and average values of each feature point along the channel dimension and concatenates them into a 2-channel feature map. We then replace the fully connected layers with convolutional layers and apply the sigmoid activation function to obtain the spatial attention coefficients. These coefficients are then used to recalibrate the feature maps that have already been recalibrated by channel attention. Specifically, this process can be represented as follows:

(9) $$\begin{array}{c} spatialattention=\mathrm{Sigmoid}\left(conv_{1d}\left(\mathrm{AvgPool}\left(F_{c}\right)\mathrm{; MaxPool}\left(F_{c}\right)\right)\right) \end{array}$$

(10) $$\begin{array}{c} F=F_{c}*spatialattention \end{array}$$

where F is the feature map after the dual calibration of channel and spatial attention.

After undergoing dual recalibration of channel and spatial attention, the feature map F highlights the most relevant EEG signal features for classification while suppressing non-essential features.

### 2.2. Feature fusion

**Figure 3:**
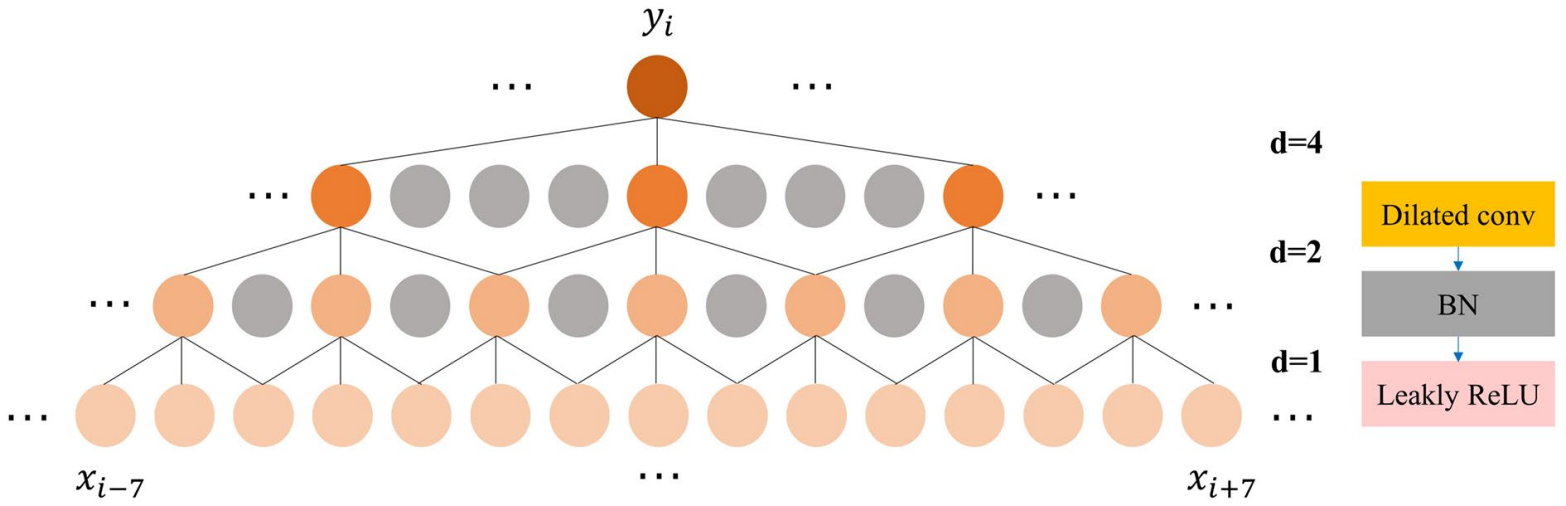
Left: The process of expanding the receptive field by the dilated convolution; Right: The composition of the dilated convolution module.

The feature extraction and adaptive recalibration module effectively extract features from the raw EEG signals. However, it is not enough to perform feature extraction alone. Feature fusion is a critical aspect of most machine learning models (Yang et al., 2003). To fuse the extracted features rapidly and efficiently, inspired by the Time Convolutional Network (TCN) (Bai et al., 2018), we incorporate the dilated convolution module. Unlike traditional convolution, which has a linear relationship between receptive field and convolutional layers, dilated convolution exponentially increases the receptive field with model depth (Yu and Koltun, 2015). To balance feature fusion with model depth, we design a three-layer dilated convolution module with a kernel size of 3. Each layer has dilation factor and stride of 1, 2, and 4, respectively, while the padding is set to 1. The number of channels is set to 32, 64, and 128, respectively. After each dilated convolution, we apply BN and Leaky ReLU activation. As illustrated in Figure 3, the output feature $y_{i}$ of the three-layer dilated convolution module fuses features from the adjacent 15 points on the initial feature map, significantly enhancing the expansion of the receptive field compared to standard convolution. The expansion of the receptive field enhances the ability to capture global contrast information, such as the location and duration of the occurrence of different sleep feature waves within one 30s EEG epoch, allowing the model to make staging decisions with a global view rather than based on local semantic information.

### 2.3. CAM

The current deep learning model is still a black box system. However, interpretability is a crucial aspect of deep learning models in the medical application. CAM is a well-established explainable model in computer vision (Zhou et al., 2016). CAM operates on the principle that the weights of the fully connected layer pertaining to the output class demonstrate the contribution of the feature map to the target class. By channel-wise multiplication and summation of the weight with the feature map, followed by upsampling to the original signal sampling rate, we obtain the model’s attention level toward the original signal under the current predicted class. The calculation process is straightforward and can be represented as follows:

(11) $$\begin{array}{c} CAM^{C}=\underset{k}{\sum }w_{k}^{C}\underset{x}{\sum }f_{k}\left(x\right) \end{array}$$

Where $f_{k}\left(x\right)$ is the activation value of the xth feature point in the kth feature map obtained through feature fusion, and $w_{k}^{C}$ is the kth weight corresponding to the output class c of the fully connected layer. By normalizing the attention level to a range of 0 to 1 and visualizing the original signal as a heatmap, we obtain the final visualization result.

## 3. Experiments

### 3.1. Data description

The proposed model is evaluated on three publicly available healthy human datasets.

The Sleep EDF dataset consists of two sub-datasets, Sleep EDF-20 and the extended version Sleep EDF-78 (Goldberger et al., 2000). The Sleep EDF-20 dataset includes overnight PSG sleep recordings from 20 healthy Caucasian individuals aged 21–35 years, with two nights of recordings for each subject except for subject 13, whose recordings are missing for one night. Therefore, this dataset includes a total of 39 sleep recordings. The Sleep EDF-78 dataset further collects two nights of PSG sleep recordings from 78 healthy individuals aged 25–101 years, except for three subjects whose recordings are missing for one night. Therefore, this dataset includes a total of 153 sleep recordings.

Each record in the Sleep EDF dataset contains scalp EEG signals from two channels (Fpz-Cz and Pz-Cz), one horizontal EOG, one EMG, and one nasal-oral respiratory signal. All EEG and EOG signals have the same sampling rate of 100 Hz. These recordings are manually classified into one of eight classes (W, N1, N2, N3, N4, REM, MOVEMENT, UNKNOWN) by sleep experts based on the R&K standard (Himanen and Hasan, 2000). We exclude all MOVEMENT and UNKNOWN stages and combine N3 and N4 stages into one sleep stage (N3), to conform to the latest AASM standard. We only use the EEG signal from the Fpz-Cz channel.

SHHS is a multi-center cohort study. Subjects have a variety of medical conditions, including pulmonary, cardiovascular, and coronary artery disease. To minimize the effects of these diseases, we follow the previous study and select subjects who are considered to have a regular sleep (e.g., Apnea Hypoventilation index or AHI less than 5) (Fonseca et al., 2017). A total of 329 individuals are screened as final experimental data. Notably, we select the C4-A1 channel with a sampling rate of 125 Hz and downsample it to 100 Hz.

To ensure a fair comparison with previous work (Eldele et al., 2021; Yubo et al., 2022; Zhou et al., 2023), we only extract data from the wake periods within 30 min before and after sleep periods. We also perform cross-validation on a per-subject basis, where the testing set consisted of data from subjects that are not included in the training set. We conduct 10-fold cross-validation on the Sleep EDF-78 dataset and SHHS dataset, and leave-one-out cross-validation on the Sleep EDF-20 dataset. Furthermore, we divide the training set into training and validation sets in each round of cross-validation. Because there may be a bias in data distribution when dividing the training set based on subject, we first shuffle the training set by epoch and then perform stratified sampling, with a ratio of 9:1 for the training set and validation set. It is worth emphasizing that the testing set is completely independent and do not participate in the stratified sampling process. Table 2 provides detailed information on the data distribution of each class in the three datasets.

**Table 2:** Details of three datasets used in our experiments.

Datasets	#Subjects	Channel	Sampling rate	W	N1	N2	N3	REM	#Total
Sleep-EDF-20	20	Fpz-Cz	100 Hz	8285	2804	17799	5703	7717	42308
				19.6%	6.6%	42.1%	13.5%	18.2%	
Sleep-EDF-78	78	Fpz-Cz	100 Hz	65951	21522	69132	13039	25835	195479
				33.7%	11.0%	35.4%	6.7%	13.2%	
SHHS	329	C4-A1	125 Hz	46319	10304	142125	60153	65953	324854
				14.3%	3.2%	43.7%	18.5%	20.3%	

### 3.2. Experiments setting and evaluation metrics

We build our model using PyTorch 1.11 and train it on a RTX 3090 GPU. It is worth emphasizing that we do not employ any preprocessing techniques, data augmentation or class balancing methods for Sleep-EDF-20 and Sleep-EDF-78 datasets. For the SHHS dataset, we apply a weighted cross-entropy loss to mitigate the serious class imbalance, with a weight matrix of [1, 2, 1, 1, 1]. We apply the Adam optimizer with a fixed learning rate and employ L2 regularization to alleviate overfitting in all experiments. The multi-class cross-entropy loss is adopted for the other two datasets. We implement early stopping if the validation loss does decrease for 10 consecutive epochs and save the model with the best validation set performance for testing. The detailed hyperparameter configurations are described in Table 3.

**Table 3:** Detailed hyperparameters for model training.

Hyperparameters	Value	Hyperparameters	Value
Batch size	200	L2 regularization	10^−3^
Learning rate	10^−3^	Early stopping	10 (valid loss)
β1 and β2	0.9, 0.999	Train epoch	100

Four metrics are adopted to evaluate the performance of sleep staging models, namely, accuracy (ACC), macro-averaged F1-score (MF1), Cohen Kappa (κ) and per-class F1 score (F1). Given True Positives (TP), False Positives (FP), True Negatives (TN), and False Negatives (FN), the overall accuracy of ACC, F1, κ are defined as follows:

(12) $$\begin{array}{c} Accuracy=\frac{TP+TN}{TP+TN+FP+FN}\times 100 \end{array}$$

(13) $$\begin{array}{c} F1=2\times \frac{Precision\times Recall}{Precision+Recall} \end{array}$$

(14) $$\begin{array}{c} \kappa =\frac{p_{o}-p_{e}}{1-p_{e}} \end{array}$$

Where $Recall=\frac{TP}{TP+FN}$ , $Precision=\frac{TP}{TP+FP}$ , the average of F1 corresponds to MF1, κ indicates the agreement between the human expert and model predict.

## 4. Results

We extensively evaluate our model on three datasets and compare it with traditional deep learning models and similar works in the field. The specific experimental results are presented in Table 4.

**Table 4:** Comparison among Micro SleepNet and baseline models.

Methods	Dataset	Parameters	Overall metrics			Per-class F1-score (F1)				
			ACC MF1 κ			W	N1	N2	N3	REM
Our method	Sleep-EDF-20	~48.2 K	82.8	75.3	0.76	86.8	35.3	87.7	89.1	77.7
DeepSleepNet	Sleep-EDF-20	~25 M	82.0	76.9	0.76	84.7	46.6	85.9	84.8	82.4
Multitask CNN (one to one)	Sleep-EDF-20	–	79.8	72.0	0.72	–	–	–	–	–
BSN 19 (MIT)	Sleep-EDF-20	–	83.5	–	–	89.0	44.0	85.0	86.0	77.0
IITNet (one to one)	Sleep-EDF-20	–	80.6	72.1	0.73	84.7	29.8	86.3	87.1	72.8
Our method	Sleep-EDF-78	~48.2 K	79.5	71.8	0.71	90.8	35.1	83.7	79.8	69.4
Sleep transformer	Sleep-EDF-78	~3.7 M	81.4	74.3	0.74	91.7	40.4	84.3	77.9	77.2
CM transformer (one to one)	Sleep-EDF-78	~320 K	78.3	–	0.70	91.4	37.7	81.6	75.3	69.3
CNN transformer	Sleep-EDF-78	~300 K	77.5	–	–	90.0	32.0	82.0	75.0	69.0
Our method	SHHS	~48.2 K	83.3	73.1	0.77	85.5	26.6	86.0	87.5	79.7

**Figure 4:**
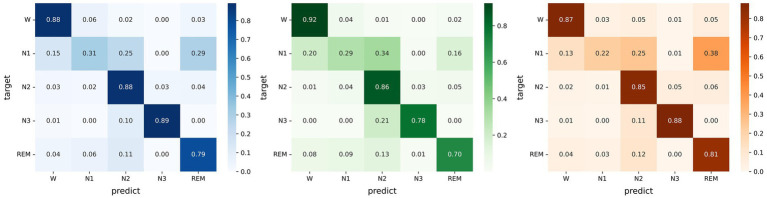
Left: Confusion matrix evaluated on the Sleep EDF-20 dataset; Middle: Confusion matrix evaluated on the Sleep EDF-78 dataset; Right: Confusion matrix evaluated on the SHHS dataset.

Micro SleepNet achieves high overall sleep staging accuracies and Cohen Kappa agreements, as presented in Figure 4. However, we observe a relatively low performance of the model in identifying the N1 sleep stage. This challenge may be attributed to the unclear waveform features of the N1 stage and the limited proportion of N1 data in the dataset could have also affected learning of the model. Moreover, the low inter-rater agreement among physicians in manually staging it, reported to be only around 63% (Rosenberg and Van Hout, 2013).

**Figure 5:**
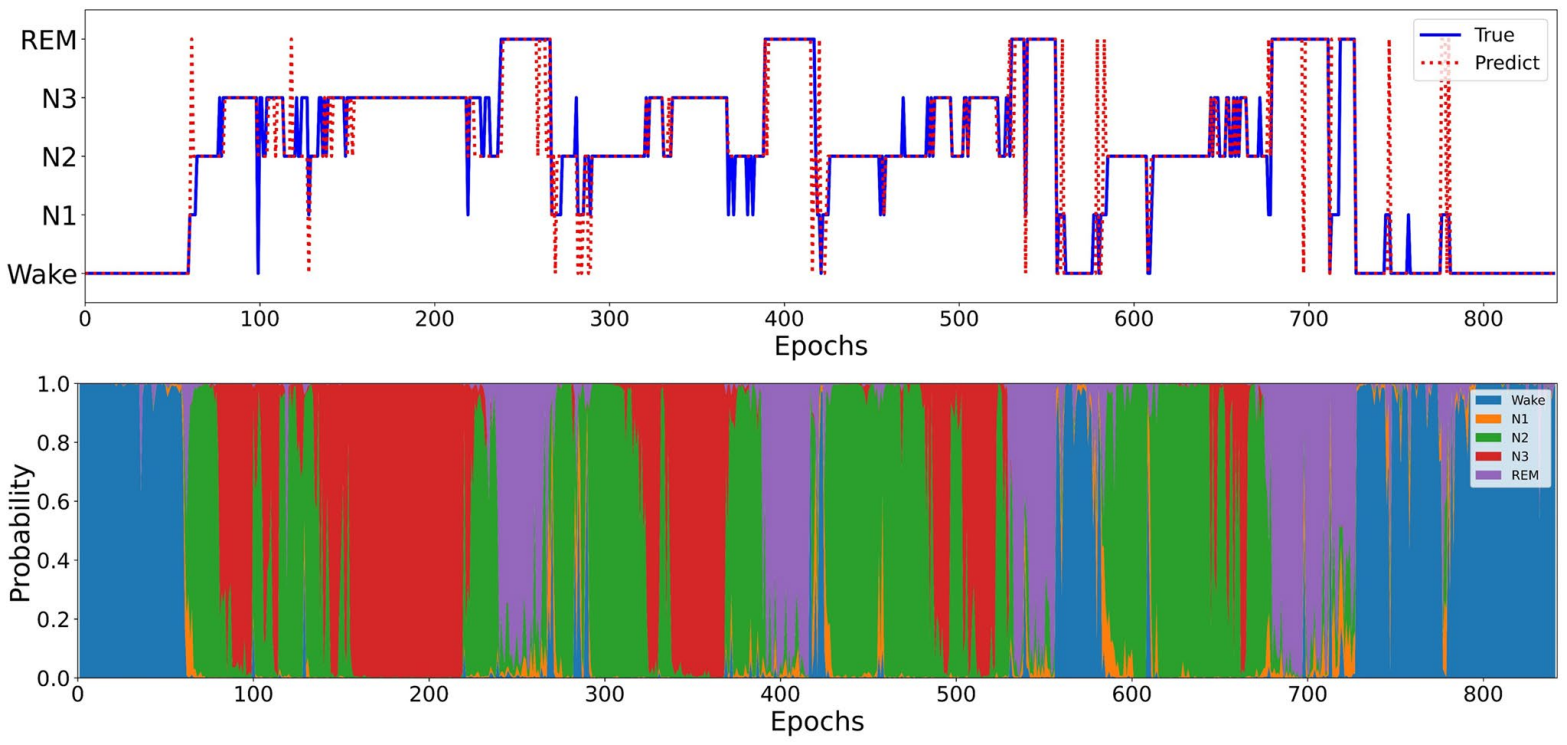
Top: One full-night hypnograms of SC4001E0 subject. The solid blue line and red dashed line denote the hypnograms depicted by a physician and the proposed model, respectively; Bottom: the posterior probability distribution over different sleep stages.

Figure 5 illustrates the sleep staging results of Micro SleepNet for the entire night of SC4001E0 data. While the model accurately classifies most of the sleep stages, it exhibits a tendency to make errors during sleep stage transitions, particularly in confusing N1 with REM sleep stage. This is a common issue in deep learning-based sleep staging algorithms, as EEG signals during the transition period exhibit features of multiple stages, making it challenging for the model to accurately classify them. Specifically, the REM sleep stage typically transitions to the N1 stage, the EEG signals located in the transition stage have the typical waves of several different periods at the same time, therefore the model is very prone to misclassification. As shown in the posterior probability distribution of each sleep stage in Figure 5, the posterior probability of the corresponding category of sleep periods in the transition stage shows a very high uncertainty. It is worth noting that even physicians encounter difficulty in accurately staging during sleep transition periods (Rosenberg and Van Hout, 2013).

### 4.1. Comparison with baselines

DeepSleepNet utilizes time-domain and frequency-domain encoding to extract raw EEG features and performs sequence modeling using BiLSTM (Supratak et al., 2017). In contrast, Micro SleepNet achieves higher overall accuracy with only 1/500 of the parameters of DeepSleepNet. In addition, the disadvantage of introducing the BiLSTM structure is that it is difficult to optimize during the training process and prone to gradient disappearance and gradient explosion. However, the F1 values of Micro SleepNet in the N1 and REM stages differs significantly from them because DeepSleepNet is modeled with sequences of length 20 and is first pre-trained with a balanced oversampled dataset. It captures temporal information during sleep stage transitions. Compared to Multitask CNN (Phan et al., 2019a), which also employs a CNN and one-to-one structure, Micro SleepNet demonstrates significantly better overall performance. One of the reasons is that Multitask CNN performs time-frequency feature extraction by performing a short-time Fourier transform on the original EEG signals, however, this may lose some useful information. Furthermore, compared to IITNet (one-to-one), which uses Resnet50 for feature encoding and two layers of BiLSTM for sequence modeling (Seo et al., 2020), Micro SleepNet outperforms IITNet in all metrics, indicating that the one-to-one LSTM structure does not provide a performance advantage and further increases parameters. BSN 19 (MIT) is the first real-time sleep staging model deployed on smartphones, using temporal convolutional neural networks for real-time inference (Koushik et al., 2019). Although the overall accuracy of Micro SleepNet is slightly lower than that of BSN 19 (MIT), yet Micro SleepNet is more lightweight and can be deployed not only on smartphones, but also directly into wearable EEG acquisition devices, making the sleep modulation system more integrated, eliminating the data transmission process and providing adequate protection of user physiological data privacy. Sleep Transformer extracts time-frequency graphs of raw EEG signals and performs sequence modeling using two layers of Transformer Encoder (Phan et al., 2022b). Consequently, Micro SleepNet still exhibits some performance gaps compared to it. However, the high parameter counts of 3.7 M and the long input sequences hinder deployment and real-time inference on mobile devices. Compared to two other relatively lightweight one-to-one CNN Transformer models (Pradeepkumar et al., 2022; Yao and Liu, 2022), Micro SleepNet achieves better staging performance with an order of magnitude fewer parameters. This result indicates that introducing Transformer structures does not significantly improve CNN model performance. Simple CNN structures, such as Micro SleepNet, can still achieve competitive results in one-to-one sleep staging tasks.

### 4.2. Ablation experiments

In order to evaluate the contribution of each module in our proposed model, we conduct ablation experiments on the SleepEDF-20 dataset. The data partition and training hyperparameters are the same as described in the previous section.

The ablation experiments are divided into five groups:

1. Gconv: only including the group convolution module;
2. Gconv + shuffle: including the group convolution module and channel shuffle;
3. Gconv + shuffle + ECSA: including the group convolution module, channel shuffle, and ECSA;
4. GSE + normal conv: using normal convolution instead of the dilated convolution module in Micro SleepNet;
5. Micro SleepNet: including all modules, Micro SleepNet.

**Figure 6:**
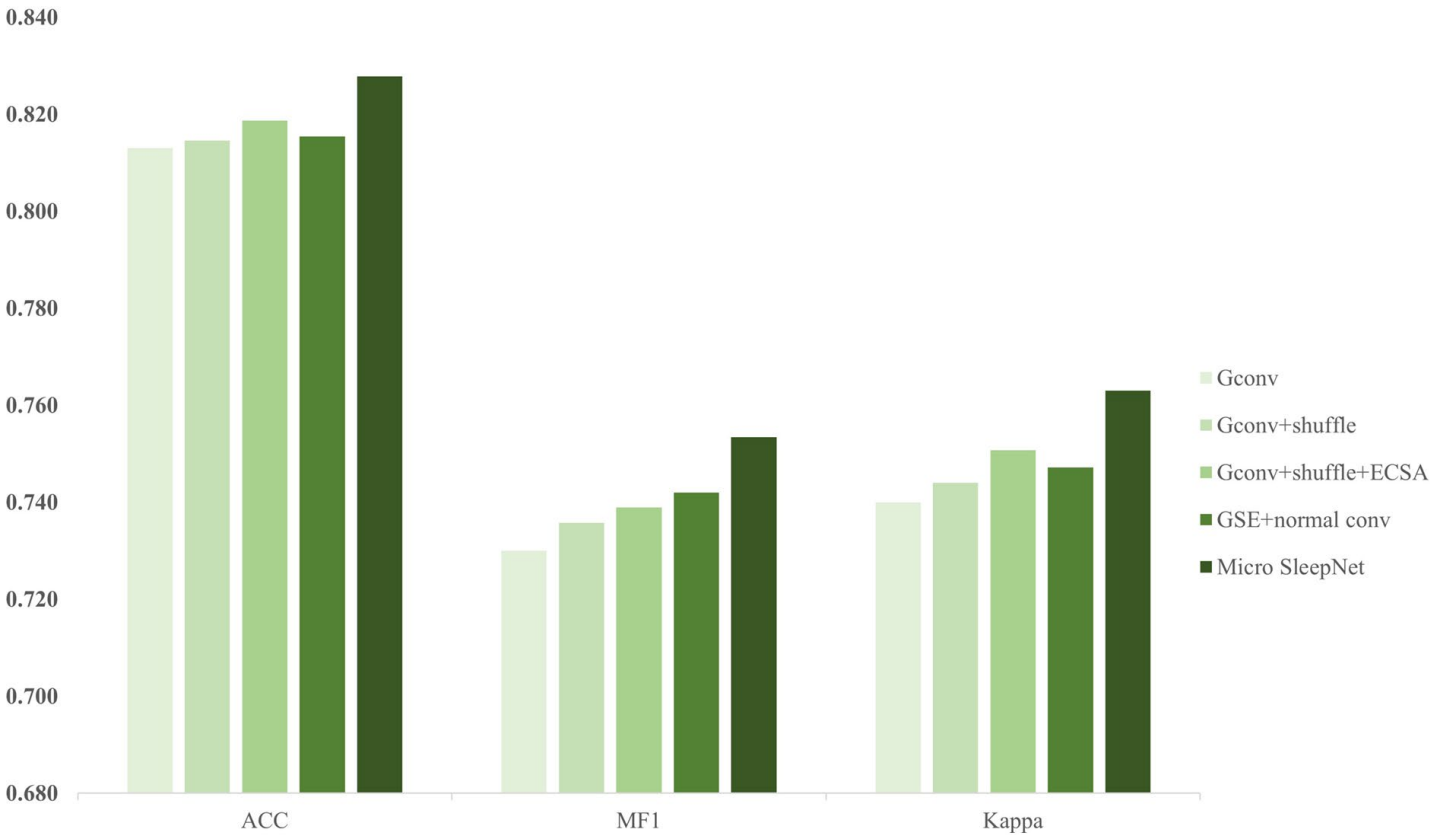
The results of ablation experiments.

Based on the results of the ablation experiments depicted in Figure 6, we discover that multiple layers of group convolution limit the representation capacity of a pure group convolution network due to inadequate information exchange between groups. However, incorporating the channel shuffle technique improves the information flow of group convolution and slightly enhances representation capacity of the model. Furthermore, the addition of the ECSA module on top of group convolution and channel shuffle further boosts the accuracy of the model by 0.5%. It is attributed to introduction of dual attention for channel and spatial dimensions by the ECSA module, resulting in adaptive feature recalibration of the feature maps generated by group convolution. The EEG feature waves that contribute more to the classification are assigned higher weights. It is worth noting that after adding the ECSA module, the number of parameters of the model only increases by 93, and the FLOPs only increases by 30.7w, indicating the efficiency of the ECSA module in improving model performance. Moreover, the control experiment of feature fusion with dilated convolution and normal convolution modules reveals that dilated convolution significantly expands the receptive field, enabling sufficient fusion of high-dimensional features extracted from the EEG signals and significantly improving classification ability of the model. In conclusion, the above ablation experiments illustrate that each module of our model contributes.

### 4.3. Architecture analysis

To verify whether the proposed model architecture achieves an optimal balance between performance and complexity, a comprehensive architectural hyperparametric analysis is performed.

From Table 5, it is shown that replacing the standard convolution with group convolution and adding GAP operations significantly reduces the computational cost and effectively avoids overfitting since GAP operations fuse the average feature values of each channel. Second, by adjusting the maximum pooling stride and the number of blocks, we find that the optimal balance between computational cost and performance is achieved when the maximum pooling stride is 3 and the number of Block layers is 5. Finally, the adjustment of the Conv kernel also indicates that a convolution kernel of size 1*3 is the most efficient. It is also consistent with the previous analysis in the paper (Simonyan and Zisserman, 2014).

**Table 5:** Detailed experimental groups and results of architecture analysis.

Methods	Parameters	FLOPs	ACC
Block*5(standard Conv) + Dconv*3 + FC	~192.2 K	~505.92 M	81.9
Block*5(MaxPool = 2) + Dconv*3 + GAP	~48.2 K	~132.17 M	81.8
Block*6(MaxPool = 2) + Dconv*3 + GAP	~49.3 K	~92.00 M	82.3
Block*6(MaxPool = 3) + Dconv*3 + GAP	~49.3 K	~38.09 M	81.6
Micro SleepNet	~48.2 K	~48.95 M	82.8
Block*4(MaxPool = 3) + Dconv*3 + GAP	~47.2 K	~81.08 M	81.9
Block*5(MaxPool = 3) + Dconv*3 + FC	~71.3 K	~49.18 M	81.6
Block*5 + Dconv*3 + GAP (kernel = 5)	~78.8 K	~68.42 M	81.3
Block*5 + Dconv*3 + GAP (kernel = 7)	~109.4 K	~87.89 M	81.9

**Figure 7:**
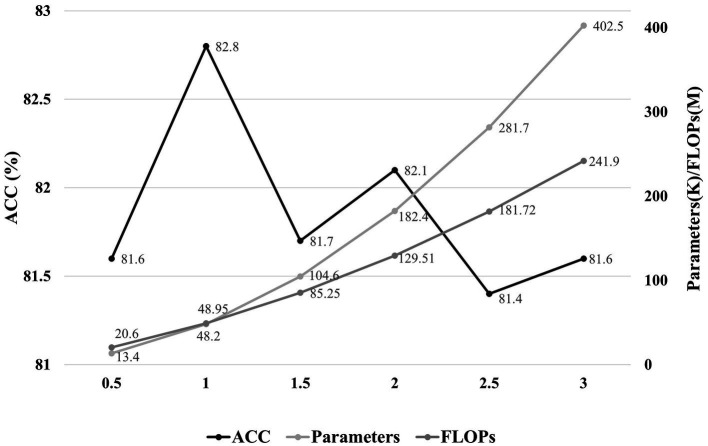
The effect of model scale on performance. The horizontal axis represents the factors of the convolution channels in each layer of the model, and the vertical axis indicates the accuracy and the parameters/FLOPs, respectively.

In addition, we also observe the effect of model scale on performance. A hyperparameter α is set as the factors for the convolution channel of each layer of the model, and we set α for the proposed model to 1. We analyze the performance and computational resources for six sets of model scale with α in the range of 0.5–3. As shown in Figure 7, the optimal balance is achieved for the model scale when α is 1.

### 4.4. Analysis of the CAM visualization

**Figure 8:**
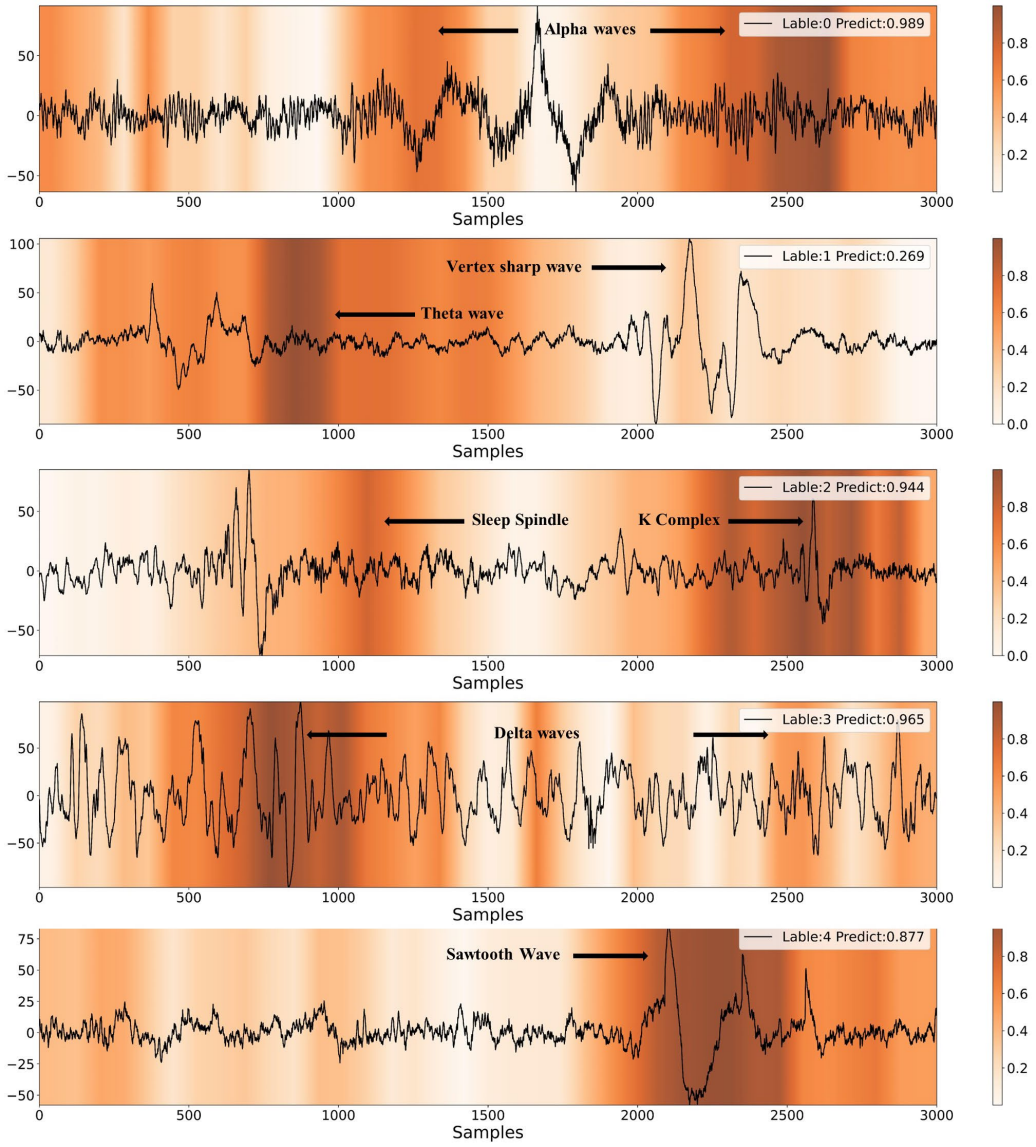
Results of CAM visualization for five different sleep stages. The labels of 0, 1, 2, 3, 4 represent W, N1, N2, N3, REM, respectively.

In Figure 8, we present heatmaps that visualize the correctly predicted classes for different sleep stages, along with the posterior probability of the predictions. It is evident that the model focuses on significantly different feature waveforms of EEG signals for different sleep stages. A detailed analysis is conducted according to AASM standards as follows.

When the EEG signal exhibits alpha rhythm for more than half of the time, sleep experts classify it as the wake stage. As shown in Figure 8, the model provides the same staging category as sleep experts. Furthermore, the majority of the alpha rhythm areas are highlighted, indicating that the model mainly identifies EEG signals as the wake stage based on alpha rhythm, which is highly consistent with the human expert. The characteristic waves of the N1 stage are theta waves (4-7 Hz) and vertex sharp waves. The model also captures theta waves and vertex sharp waves. In the N2 stage, the EEG signal usually contains one or more K-complexes and sleep spindles. It can be observed that the model emphasizes both K-complexes and sleep spindles. If more than 20% of the EEG signal shows slow-wave activity, it can be classified as the N3 stage. The model highlights the slow-wave activity area when making N3 stage predictions. The typical waveform of REM stage EEG is the sawtooth wave, which the model accurately identifies.

The analysis indicates that the model uses typical feature waves of different sleep stages to conduct sleep staging, which highly corresponds to the interpret process by human expert. Therefore, the model has learned the AASM staging criteria based on high-quality data annotated by experts and has a considerable degree of interpretability, providing strong evidence for the rationality of the model structure design. Furthermore, the combination of the model and CAM technology is expected to assist the sleep staging process by guiding doctors to quickly focus on sleep feature waves.

## 5. Discussion

Due to the lack of precise real-time sleep tracking, previous sleep modulation has been open-loop, unable to adjust the stimulation methods and parameters based on the real-time sleep stage (Marshall et al., 2004; Bellesi et al., 2014; Perl et al., 2016). This open-loop sleep modulation method has significant limitations. To achieve complete closed-loop sleep modulation, it is necessary to develop a real-time sleep staging algorithm that satisfies the deployment conditions of mobile devices.

**Figure 9:**
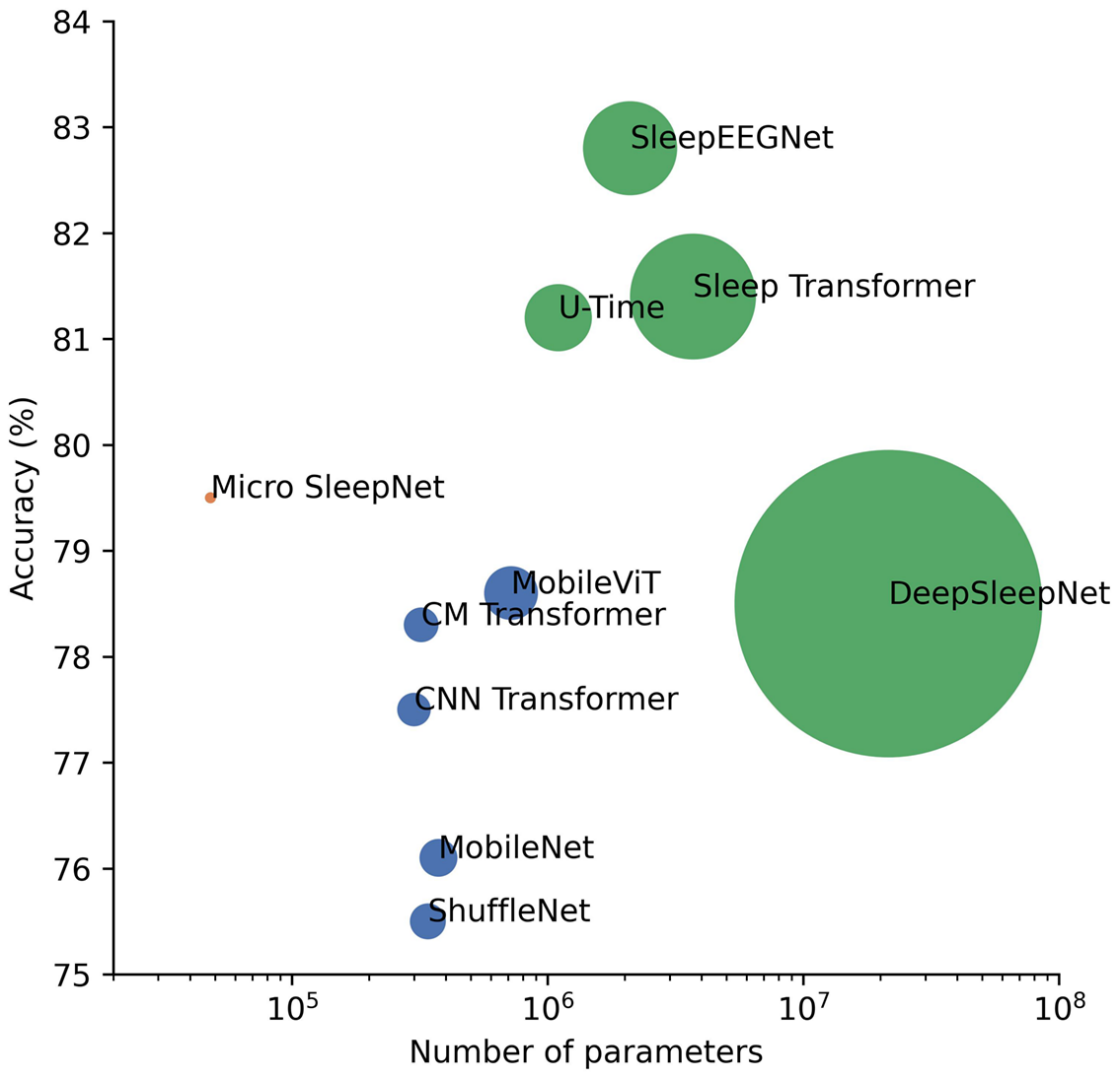
Plot of performance versus number of parameters for partial sleep staging models as well as lightweight models in computer vision on the Sleep-EDF-78 dataset. The green dots represent the time-series model, the dark blue dots represent the non-time-series model, and the pink dots represent the proposed model. The size of the dots is a linear relationship with the number of parameters of the model.

In this paper, we propose a lightweight deep learning model named Micro SleepNet, specifically designed for real-time sleep staging on mobile devices. Unlike most traditional deep learning sleep staging models, Micro SleepNet does not rely on contextual temporal signals and only uses the current input EEG signal for sleep staging. It uses one-dimensional group convolution and extremely lightweight ECSA module for efficient feature extraction, efficient feature fusion using dilated convolution, and greatly reduces parameters and computational complexity of the model. Thanks to the efficient model design, it achieves competitive performance with significantly fewer parameters than traditional deep learning models, with an overall accuracy of 83.3% and the Cohen Kappa is 0.77 on the SHHS dataset. Additionally, we introduce CAM for the first time to the EEG sleep staging field, and the visualization results show that the model performs sleep staging based on different EEG feature waveforms during different periods, highly consistent with the staging process of the human expert, providing a solid interpretability foundation for future clinical applications. In addition, the combination of the model and CAM is expected to assist junior physicians in artificial sleep staging. Finally, a series of architectural analyses and ablation experiments show that the architecture of the model achieves an optimal balance between performance and computational resources, and each component contributes to the performance improvement, supporting the rationality of the model design. We also compile a plot of accuracy versus number of parameters for partial models on the Sleep-EDF-78 dataset (Sandler et al., 2018; Mousavi et al., 2019; Mehta and Rastegari, 2021). As shown in Figure 9, our model is optimal in the non-time-series model. It is owing to efficient model architecture design.

Furthermore, we deploy the model on the Android platform based on NCNN framework of Tencent. The RAM and ROM of the Android platform are 8 GB and 256 GB, respectively. The results show that the model file occupies approximately 100 KB of memory, and the inference time for each data based on CPU on the Qualcomm Snapdragon 865 processor is only 2.8 ms, with the inference results identical to those on the PC side. This verifies the feasibility of edge computing implementation on mobile devices, and the model can perform real-time sleep staging tasks for wearable health monitoring scenarios, supporting the implementation of high-precision real-time assisted sleep and sleep closed-loop modulation systems. In total, our work provides a new way of thoughts for mobile sleep staging model design.

Although the proposed method achieves competitive performance on three publicly available healthy subject datasets, the behavior of the model on other larger and more diversity datasets is still uncertain, thus the robustness on different datasets needs to be further validated. Considering the actual usage scenario of the model, in the future, a large-scale sleep dataset of people with mild sleep disorders should be collected to evaluate the performance of model more in line with the actual population. In addition, we do not choose the model design idea of temporal modeling because even though it has been widely demonstrated in the paper that doing so significantly improves model performance (Olesen et al., 2020, 2023; Phan et al., 2023), on some MCUs with limited storage resources, storing a large number of historical signals is not feasible and would additionally increase the computational burden. It is part of the limitation of our study that this model is not specifically designed for deployment on smartphones. However, for models designed to be deployed on smartphones, it would be preferable to do so, as the performance of sleep staging is significantly improved by integrating the temporal information of the historical signal with that of the current moment, and the magnitude of the improvement will depend on the length of the integrated historical temporal signal. Also doing so subtly ensures real-time efficiency. U-time may be a potentially suitable model architecture (Perslev et al., 2019). Moreover, 5-classification experiments are performed in this study, and yet in some sleep modulation scenarios four or even three classification can satisfy the requirements (Nguyen et al., 2022; Koyanagi et al., 2023), and different modulation methods focus on the real-time detection of different sleep stages (Ngo et al., 2013; Lu, 2020; Nguyen et al., 2022), hence further customization of the algorithm for specific modulation methods will be the direction of future work. In addition, this study only verifies the model deployment on the Android smartphone. In the future, the model should be further deployed on wearable EEG acquisition devices to verify whether the performance indicators for inference at the acquisition end meet the requirements of actual usage scenarios. Finally, due to the significant differences in the distribution of actual EEG signals collected on mobile devices compared to publicly available healthy PSG data (Heremans et al., 2022) and the generally inconsistent acquisition channels with PSG acquisition channels, it is difficult for professional physicians to interpret wearable EEG data. We consider using unsupervised domain adaptation methods to overcome domain mismatch when collecting unlabeled actual EEG data on mobile devices.

## Data availability statement

Publicly available datasets are analyzed in this study. The Sleep-EDF can be found in https://www.physionet.org/content/sleep-edfx/1.0.0/, and the SHHS can be found in https://sleepdata.org/datasets/shhs.

## Author contributions

GL, JZ and DZ: conceptualization and methodology. DM: data curation. XT, XW and NC: funding acquisition and supervision. GL: investigation, software, and writing—original draft. NC and XW: project administration. GL, JZ, DM, GW, SS, XW, and NC: writing—review and editing. All authors contributed to the article and approved the submitted version.

## Funding

This work was supported by the National Natural Science Foundation of China Youth Fund (Grant No. 31700856) and the National Key Research and Development Program of China (2020YFC2007903).

## Conflict of interest

The authors declare that the research was conducted in the absence of any commercial or financial relationships that could be construed as a potential conflict of interest.

## Publisher’s note

All claims expressed in this article are solely those of the authors and do not necessarily represent those of their affiliated organizations, or those of the publisher, the editors and the reviewers. Any product that may be evaluated in this article, or claim that may be made by its manufacturer, is not guaranteed or endorsed by the publisher.
